# Dynamic Navigation and Area Assignment of Multiple USVs Based on Multi-Agent Deep Reinforcement Learning

**DOI:** 10.3390/s22186942

**Published:** 2022-09-14

**Authors:** Jiayi Wen, Shaoman Liu, Yejin Lin

**Affiliations:** Lab of Intelligent Marine Vehicles of DMU, Dalian Maritime University, Dalian 116026, China

**Keywords:** USV, trajectory design, policy gradient, multi-agent deep reinforcement learning, multi-object optimization

## Abstract

The unmanned surface vehicle (USV) has attracted more and more attention because of its basic ability to perform complex maritime tasks autonomously in constrained environments. However, the level of autonomy of one single USV is still limited, especially when deployed in a dynamic environment to perform multiple tasks simultaneously. Thus, a multi-USV cooperative approach can be adopted to obtain the desired success rate in the presence of multi-mission objectives. In this paper, we propose a cooperative navigating approach by enabling multiple USVs to automatically avoid dynamic obstacles and allocate target areas. To be specific, we propose a multi-agent deep reinforcement learning (MADRL) approach, i.e., a multi-agent deep deterministic policy gradient (MADDPG), to maximize the autonomy level by jointly optimizing the trajectory of USVs, as well as obstacle avoidance and coordination, which is a complex optimization problem usually solved separately. In contrast to other works, we combined dynamic navigation and area assignment to design a task management system based on the MADDPG learning framework. Finally, the experiments were carried out on the Gym platform to verify the effectiveness of the proposed method.

## 1. Introduction

Nowadays, various kinds of unmanned robots are developing rapidly with the arrival of the 5G era. Typical robots include the autonomous underwater vehicle (AUV), unmanned surface vessel (USV), unmanned ground vehicle (UGV), and unmanned aerial vehicle (UAV) [1,2,3,4], which play important roles in the artificial intelligence (AI)-enabled next-generation (6G) network. As an important unmanned marine tool, the USV is also a promising technique to provide wireless communication due to its low cost and high flexibility [5,6]. Especially when it comes to complex tasks, such as maritime joint search and rescue, maritime multi-target search, the construction of marine information networks, and other missions. However, it is difficult to rely on one single USV to complete the task, while multi-USV cooperation can be a good solution to the problem. This paper studies the multi-USV dynamic path planning and area assignment problem, which will effectively improve the autonomy and reliability of the USV in the increasing field.

As a generic technology among different robotics systems, the optimization objectives mainly include path length, time or energy consumption, risk measure, maneuverability, etc. They are classified into four kinds based on the model of consumption space, i.e., the grid-based methods [7,8,9,10] solved by the heuristic strategies, such as the A* algorithm, D* algorithm, Dijkstra algorithm, and Q-learning algorithm in reinforcement learning theory, or intelligent strategies represented by particle swarm optimization and evolutionary algorithms; the sampling-based methods such as probabilistic roadmap, rapidly-exploring random tree (RRT) [11,12,13], or deep reinforcement learning method represented by experience replay based DQN algorithm; the mathematical optimization methods such as mixed integer linear programming (MILP) and model predictive control (MPC) [14,15,16]; and the potential field methods such as the artificial potential field (APF) and the interfered fluid dynamical system (IFDS) [17,18,19].

In addition to the classification mentioned above, when the purpose of agents is taken as the dividing boundaries, the path planning problem is divided into the single agent path planning problem (SAPP) and the multi-agent path planning problem (MAPP). For the requirement of accessing a series of intermediate points during the monitoring mission in SAPP, Ning Wang et al. [20] proposed a successive waypoints tracking method using a BT-guided model-free solution to solve the SWT problem. Yong et al. [21] proposed the dynamic augmented multi-objective particle swarm optimization algorithm to solve the problem that obstacles and water flows exist at the same time to find the shortest and safest route to the target, which is subject to collision avoidance. The rapidly random-exploring tree (RRT) algorithm and its variants are some of the more popular path planning methods in SAPP. However, they suffer sensitivity to the initial solution, which requires a lot of memory and time to converge to the optimal solution. In order to solve this problem, Wang et al. [22] propose the NRRT* to achieve nonuniform sampling in the path planning process by learning quantities of successful planning cases from the A* algorithm. Thus, the sampling process is guided, and the efficiency of the algorithm is improved. The sampling mechanism is also used in reinforcement learning which has been prevalent in recent years. In [23], Tom Schaul et al. proposed the prioritized experience replay method to speed up the convergence of the training process, and the effectiveness was verified on the Gym platform.

For the multi-objective optimization problem in SAPP, Hongqiang Sang et al. [24] proposed a novel deterministic algorithm named the multiple sub-target artificial potential field (MTAPF) based on the heuristic A* algorithm. The optimal path is divided by this algorithm into multiple sub-target points to form a sub-target point sequence. Ning Wang et al. [25] proposed a multilayer path planner (MPP) with global path-planning (GPP), collision avoidance (CA), and routine correction (RC) for an unmanned surface vehicle (USV). In addition, some methods inspired by these problems or traditional search strategies are suggested, such as the bridge access path-planning method [26,27].

However, it is difficult to complete tasks only by relying on one single agent in certain scenarios, such as maritime joint search and rescue, maritime multi-target search, the construction of the marine information network, etc. Thus, the MAPP approach becomes another effective method to deal with complex multi-objective problems. Yu Wu et al. [28] proposed a new cooperative path planning algorithm based on an improved particle swarm optimization (IPSO) algorithm aimed at maximizing the search space, minimizing the terminal error, and generating paths in a centralized or distributed mode. In the same way, Pradhan B. et al. [29] realized multi-robot navigation tasks by combining particle swarm optimization (PSO) with the feed forward neural network (FFNN). To optimize search capability, Yu Wu et al. [30] proposed a clustering improved ant colony optimization (CIACO) algorithm, which strengthens the global and local search ability in the early and later phases of iterations. Xinghai Guo et al. [31] proposed a chaotic and sharing-learning particle swarm optimization (CSPSO) algorithm. The path planning problem is divided into two stages: global path planning and path control, to solve the extended TSP, and the nonlinear multi-objective model. For the multi-objective joint optimization problem, Milad Nazarahari et al. [32] proposed an enhanced genetic algorithm (EGA) to improve the initial paths in continuous space and jointly optimize the path length, smoothness, and safety of the agent.

However, the alternating algorithm may not converge as the number of optimized variables of USV is increased [33]. In addition, the optimized results can only be used for the current environment, while, when the environment changes, the proposed optimization algorithms can become invalid.

Deep reinforcement learning (DRL), as a branch of artificial intelligence (AI), provides an alternative solution for such complex optimization problems, such as resource allocation for V2V communications [34], the stochastic shortest path (SSP) problem [35], and mode selection and resource allocation for the fog radio access networks [36]. The DRL method can deal with a large state space and time-varying environments [37].

However, in the multi-agent reinforcement learning scenario, each agent is unstable and the environment is changing. In this paper, we propose to use the multi-agent deep deterministic policy gradient (MADDPG) algorithm to solve the area assignment and dynamic trajectory design problem in a multi-USV cooperation task. The algorithm utilized centralized training within the decentralized execution framework [38]. Each USV can be regarded as an agent. The contributions of this paper can be summarized as follows:

- We propose a multi-agent DRL method for the joint optimization problem in the multi-USV cooperation scenario, where they share the common reward function to achieve the maximum success rate of the system. The number of UAVs can be arbitrary in the proposed MADRL algorithm, while the conventional methods can only deal with the simple case, i.e., no more than two USVs;
- We consider a scenario in which obstacles change position at fixed intervals and the USV needs to adjust its actions in real-time to avoid collisions with obstacles and other USVs, ultimately achieving the task of dynamic trajectory design and area assignment. Our algorithm is designed for 2D space, in which the trajectory of the UAV can be shown in 2D;
- We develop an efficient task management framework, which adopts centralized training and the decentralized execution method. In order to improve the learning ability of the agent and better adapt to the environment, we add the “soft update” mechanism in MADDPG, so that the target network can better track the learning policy. All USVs work in a cooperative way to achieve the reasonable allocation of target areas. In order to maximize cooperative rewards, all experiences are trained together, while all behaviors are at the disposal of the USV itself. The output of the actor network is the action of the USV, which is based on the USV’s own observation.

The rest of the paper is organized as follows: In Section 2, we present the system model and problem formulation. Section 3 demonstrates the MADDPG method for the cooperative multi-USV network. Section 4 shows the simulation results and discussions. Finally, Section 5 elaborates on the conclusion of this paper.

## 2. System Model

**Figure 1:**
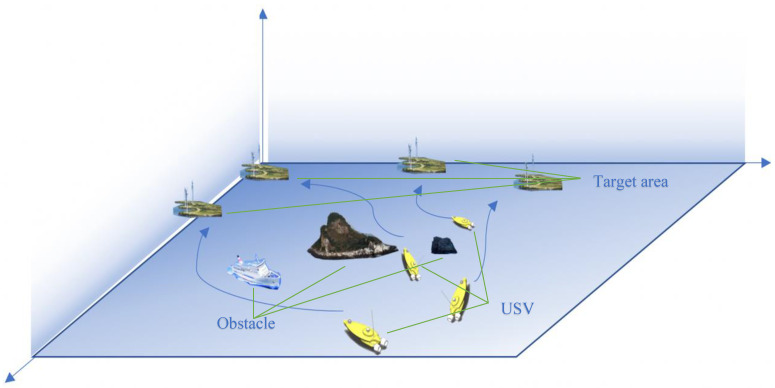
Multi-USV communication system.

This paper considered a multi-USV cooperative dynamic navigation and area allocation scenario with random obstacles on the sea surface, K USVs, N obstacles, and K target areas, as shown in Figure 1. Each USV departs from the same point and adjusts the target area to be reached according to the safety of the surrounding environment and the distance required to reach the target area. Specifically, each USV should keep a certain distance from other USVs during navigation to ensure communication as well as safety. At the same time, when encountering obstacles, it can smoothly avoid obstacles under the premise of keeping the maximum communication distance with other USVs. In order to simulate the real dynamic port situation, obstacles involved dynamic obstacles and static obstacles, among which dynamic obstacles would change their positions randomly after each training episode.

Next, we will describe the multi-USV system and the problem formulation for the proposed multi-USV cooperative dynamic navigation and area allocation scenario.

### 2.1. Multi-USV System

We considered a two-dimensional environment in which K USVs were represented by U, each USV’s position at time t is denoted by $\left(x_{i}^{U}(t),y_{i}^{U}(t)\right)$, and the matching target is represented by $T_{i}$, among which the obstacle is denoted by $O_{i}$, and the positions of targets and obstacles are represented by $\left(x_{i}^{T},y_{i}^{T}\right)$ and $\left(x_{i}^{O},y_{i}^{O}\right)$, respectively. For an arbitrary USV, the path taken can be denoted by $P_{i}=\left\{\left(x_{i}^{U}(0),y_{i}^{U}(0)\right),\left(x_{i}^{U}(1),y_{i}^{U}(1)\right),\ldots ,\left(x_{i}^{U}(n),y_{i}^{U}(n)\right)\right\}$ and the path length by $\sum_{i}^{K_{U}}d_{i}$. Thus, the discrete dynamics model of each USV can be expressed as:

(1) $$\left\{\begin{array}{c} v_{i}^{t+1}=v_{i}^{t}+\frac{F_{i}^{t}}{m}\Delta t\\ p_{i}^{t+1}=p_{i}^{t}+v_{i}^{t}\Delta t \end{array}\right.$$

where $v_{i}^{t}$ and $p_{i}^{t}$, respectively, represent the speed and position of the $i_{th}$ USV at time t, and Δt represents the sampling period where F is a force vector. The distance between the USV and the obstacle is represented by $d_{i}^{O}$, and the distance between USVs is represented by $d_{i}^{U}$, while the distance from the USV to the matching target is represented by $d_{i}^{T}$.

### 2.2. Problem Formulation

In the given system model, our goal is to enable all USVs to autonomously navigate to their respective target areas by optimizing all trajectories, and each target is only assigned to one USV. Then, we have

(2) $$\left\{\begin{array}{l} \overset{K_{U}}{\underset{i=1}{\cup }}T_{i}^{\prime }=T,i\in \left\{1,\ldots ,K_{T}\right\}\\ \forall i\neq j,T_{i}^{\prime }\neq T_{j}^{\prime }\;i,j\in \left\{1,\ldots ,K_{T}\right\} \end{array}\right.$$

where T represent all assignable regions. All USVs can navigate by adjusting their positions dynamically to avoid static and dynamic obstacles, which can be expressed as

(3) $$\forall i,j,O_{j}\notin P_{i}\;i\in \left\{1,\ldots ,N\right\},j\in \left\{1,\ldots ,U_{K}\right\}$$

During the mission, each USV needs to maintain a certain distance to stay within communication distance. We utilized Z to describe the communication graph of K USVs, where E is the edge set, defined as

(4) $$E=\left\{\left(U_{i},U_{j}\right)|d\left(r_{i},r_{j}\right)=p_{i}-p_{j}\leq \rho_{c}\right\}$$

where $\rho_{c}$ is the maximum communication distance. In other words, when the distance between two USVs is less than the maximum communication distance, there will be an undirected edge between them, and they can communicate with each other. Accordingly, each USV collaboratively adjusts its position and selects the target area based on the hazards of its environment. The collision-free path is then formulated as:

(5) $$\forall i,j,\left(x_{i}^{U}(t),y_{i}^{U}(t)\right)\neq \left(x_{j}^{U}(t),y_{j}^{U}(t)\right)$$

Thus, in this article, a multi-agent deep reinforcement learning-based trajectory design and area assignment algorithm are proposed.

## 3. MADDPG Approach for Cooperative Multi-USV Network

The multi-USV collaboration optimization issue is challenging as it requires a joint kinetic USV trajectory and area assignment. In fact, this is NP-hard, and the approach of exhaustion is usually invalid for such a multiple USVs scenario. As the quantity of USVs increases, the calculation intricacy will elevate remarkably. As far as we know, there is research utilizing the traditional optimization algorithm to tackle this problem in these intricate settings. The MARL can solve this dynamic multi-objective optimization problem effectively and accurately. Therefore, in this section, we will delineate how to utilize the MARL approach to tackle the multi-USV collaboration issue. In Section 3.1, we formulate the proposed problem as a Markov process and define the agent, state, action, and reward separately. Section 3.2 presents the MADDPG algorithm for the multi-USV joint optimization scenario.

### 3.1. Markov Game for Multi-USV Cooperation

To study the best behavior mode in given states, we employ a Markov decision process (MDP) [39] to formulate the AUV motion planning, as the Markov decision process (MDP) offers a mathematical framework for simulating the randomized strategies that can be implemented with Markov states in a given scene. The method is modeled as tuples (S,A,R,P,γ), and in this case, the tuples are action set A, state set S, state transition probability P, discount factor γ, and reward function R of the system. The action selection of the agent, a functional mapping from each state $s_{i}^{t}\in S$ to action $a_{i}^{t}\in A$, is modeled as policy π. The value function $v_{\pi }(s)$ of the process is defined as the expected sum of discount rewards that act continuously from and along with state s. In case a policy π can attain the best from any initial state $s_{i}^{t}\in S$, we detect it as an optimal policy $\pi^{*}$. This is the target of this training of the deep neural network with a deep reinforcement learning module.

**Figure 2:**
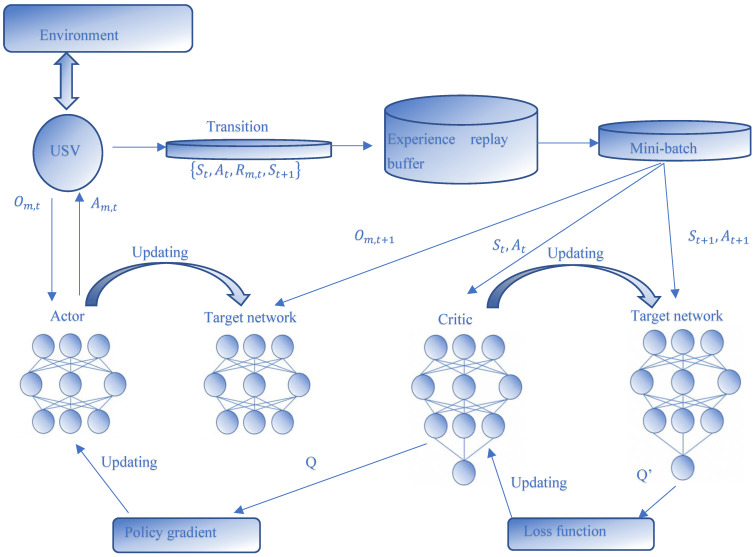
The framework of MADDPG for the cooperative multi-USV network.

Agent: Each USV can be considered an agent. USVs maintain a certain distance from each other during navigation to facilitate communication, and each USV obtains a state containing its own information and the information of the surrounding environment in the process of interaction with the environment. The environment in this problem is completely observed; therefore, the observations are equivalent to the state. Each actor has its own actor and critic network which act as the executor and evaluator of the policy, respectively. Each agent observes its own state, takes actions according to its own strategy, and then obtains rewards from the environment to reach the next state, as shown in Figure 2.

State: The state of any agent is a one-dimensional vector with five components, $\left[x,y,d_{i}^{U},d_{i}^{O},d_{i}^{T}\right]$. The first two elements represent the current 2D position of the agent, the third entry $d_{i}^{U}$ represents the distance between agents, and the fourth entry $d_{i}^{O}$ represents the distance between agent i and the surrounding obstacles. The fifth element $d_{i}^{T}$ represents the distance from agent i to the current matching target which can be changed with the changes in the USV’s selection.

Action: The action for each agent is the output of its actor network, which is expressed by $\nu_{i}=u_{i}\left(o_{i}\right)$. This is a Gaussian distribution where $v_{i}$ is composited by the velocities of the x and y axes. An action is executed by the formula $\vec{a}=\vec{v}*\Delta t+N_{t}$, where $\vec{a}$ is a displacement vector and $N_{t}$ is a random noise. The action is determined by the position of the target area and the risk factors of the surrounding environment.

Reward: Each USV obtains its own reward, which depends on the current state, current actions, and the next state $\left(s,a,s^{\prime }\right)$ at each time slot t. In our proposed multi-USV scenario, the reward consists of four parts: communication distance $l^{\max}$ limitation penalty, distance penalty, threat area penalty, and collision penalty. We utilized $R_{l}$ to represent the communication limitation reward, which depended on the next state of the agent. There will be a large minus penalty $-R_{l}$ for the agent if the agent is out of the communication space in the next state. The space limitation is set as follows

(6) $$0\leq X_{k,t}\leq l^{\max},\forall k\in K,t\in T$$

and

(7) $$0\leq Y_{k,t}\leq l^{\max},\forall k\in K,t\in T$$

where $X_{k,t}$ and $Y_{k,t}$ represent the horizontal and vertical coordinates of USV, respectively. In the same way, we denote $R_{k,o}$ as the collision penalty, which can be described as

(8) $$\left\{\begin{array}{l} R_{k,o}=-\sum_{k=1}^{K}z_{k,o,t}P_{k,o}(t)\\ z_{k,o,t}=\{0,1\},\forall k\in K,o\in O,t\in T \end{array}\right.$$

where $z_{k,o,t}=1$ means there has been a collision, and $R_{k,o}(t)$ is the collision penalty of USV. Additionally, we set up a threat area penalty $R_{k,r}$ to maintain a certain communication distance between the USVs and prevent collisions. Then, we have

(9) $$R_{k,r}={\;d}_{\min\left(u_{i},u_{j}\right)}+\sigma -{\;d}_{u_{i}^{t},u_{j}^{t}}$$

where $d_{\min\left(u_{i},u_{j}\right)}=\;\mathrm{agent}.\mathrm{size}\;+\;\mathrm{agent}.\mathrm{size}$, σ represents the width of critical area of agents, and $d_{u_{i}^{t},u_{j}^{t}}$ represents the distance between agents. In order to guide the USV closer to the target, we give the USVs a reward dependent on the distance between the USV and the target as

(10) $$R_{k,t}=\sum_{k=1}^{K}\frac{k_{dis}}{\sqrt{{\left(x_{i}-x_{t}\right)}^{2}+{\left(y_{i}-y_{t}\right)}^{2}}}$$

where $R_{k,t}$ is the distance reward for USV, and $\left(x_{i}-x_{t}\right)$, $\left(y_{i}-y_{t}\right)$ are the coordinates of the USV. Moreover, $k_{dis}$ is the hyperparameters set manually. Thus, the whole reward $R_{w,t}$ is

(11) $$R_{w,t}=R_{l}+R_{k,o}+R_{k,r}$$

### 3.2. Multi-Agent DDPG Approach

In the collaborative MARL scenario, each agent engages in interactions with the environment and acquires team rewards to facilitate collaboration. Single-agent reinforcement learning algorithms, such as proximal policy optimization (PPO) and the deep Q network (DQN), can be straightly utilized to deal with collaborative scenarios by letting each agent learn its optimum Q function in an independent manner. Nevertheless, the environment can be unstable from the perspective of any agent. To tackle the unstable issue in MARL, we utilized the MADDPG algorithm [38] learning a centralized Q-function for each agent as per the global information. When all the actions of the agents are known, the environment is stable. This is due to

(12) $$P\left(s^{\prime }|s,a_{1},\ldots ,a_{L},\pi_{1},\ldots ,\pi_{L}\right)=P\left(s^{\prime }|s,a_{1},\ldots ,a_{L}\right)=P\left(s^{\prime }|s,a_{1},\ldots ,a_{L},\pi_{1}^{\prime },\ldots ,\pi_{L}^{\prime }\right)$$

for any $\pi_{i}\neq \pi_{i}^{\prime }$, in which $\pi_{i}$ is the policy of agent i.

The aim of each agent is to select the beneficial policy that maximizes the accumulated reward $J(\theta )=E_{s\sim p^{\pi },a\sim \pi_{\theta }}[R]$ prior to the description of the MADDPG arithmetic. Let us start with the policy gradient (PG) algorithm utilized for the continuous control issue. The crucial goal of the PG is to straightly adjust the parameter θ of policy π at the orientation of $\nabla_{\theta }J(\theta )$, which is expressed as

(13) $$\nabla_{\theta }J(\theta )=\int_{S}\rho^{\pi }(s)\int_{A}\nabla_{\theta }\pi_{\theta }(a|s)Q^{\pi }(s,a)dsda=E_{s\sim p^{\pi },a\sim \pi_{\theta }}\left[\nabla_{\theta }\log\pi_{\theta }(a|s)Q^{\pi }(s,a)\right]$$

where the policy $\pi_{\theta }$ is stochastic. At each step, the action is sampled as per the conditional probability density $\pi_{\theta }\left(a|s\right)$. When the action space dimension is very large, the PG algorithm may need more samples, which will induce a remarkable computational challenge. Unlike the random policy requiring the exploration of the full state and action space in (12), the deterministic policy gradient (DPG) takes a deterministic policy into consideration $\mu_{\theta }:S\to A$, which merely needs to integrate over the state space, as

(14) $$\nabla_{\theta }J(\theta )={\left.\int_{S}\rho^{\mu }(s)\nabla_{\theta }\mu_{\theta }(s)\nabla_{a}Q^{\mu }(s,a)\right|}_{a=\mu_{\theta }(s)}ds=E_{s\sim p^{\mu }}\left[{\left.\nabla_{\theta }\mu_{\theta }(s)\nabla_{a}Q^{\mu }(s,a)\right|}_{a=\mu_{\theta }}(s)\right]$$

It is obvious from (14) that the DPG arithmetic prevents the integral over the entire action space, which can decrease the computational intricacy and ameliorate the training efficiency. In contrast to PG, DPG can tackle the difficult enhancement issue with high-dimensional actions.

As the extension of DPG, DDPG adopts the deep neural network (DNN) to approximate the policy μ and critic $Q^{\mu }\left(s,a\right)$. Nevertheless, the updated network $Q^{\mu }\left(s,a|\theta^{Q}\right)$ cannot be straightly utilized for estimating the target value, which will induce the update Q divergent. Hence, the DDPG algorithm utilizes the soft target updates rather than straightly copying the weights of the update Q.

Specifically, DDPG utilized a copy of the actor and critic networks to calculate the target value, which is denoted as $Q^{’}\left(s,a\right)$ and $\mu_{\theta }^{’}(s)$, respectively. For the sake of suiting the environment and ameliorating the exploration efficiency, another trick of DDPG is to add stochastic noise into the actor policy, as presented in Algorithm 1.
**Algorithm 1.** Training algorithm using the MADDPG framework.**Parameters:** batch size β, training episodes. M, training step T, action noise N and the number of USVs N, actor networks’ weights for each agent $\theta_{i}^{\mu^{’}}$
1: **For** episode = 1 to M **do**2:  Initialize observations $O_{\mathrm{init}\;}$, $O_{\mathrm{new}\;}\leftarrow O_{\mathrm{init}\;}$.    **Initialize:** Actor network μ, critic network Q with weights $\theta^{\mu }$, $\theta^{Q}$.    **Initialize:** Target actor, critic network: $\mu^{\prime }$, $Q^{\prime }$, with weights $\theta^{\mu^{\prime }}\leftarrow \theta^{\mu }$, $\theta^{Q^{\prime }}\leftarrow \theta^{Q}$.    **Initialize:** Replay buffer D with capacity C, exploration counter. Counter =0
3:  **for** step t=1 to T **do**4:   **if**
Counter< C **then**5:    each USV i randomly chooses $a_{i}$;6:7:    **else**8:     $a_{i}=\mu_{\theta_{i}}\left(o_{\mathrm{new}\;}^{i}\right)+N$
9:    **end if**
10:    Execute actions $a=\left[a_{1},a_{2},\ldots a_{N}\right]$, and observe reward R, new states $o_{new}$;11:    Store transition $\left(o,a,r,o_{\mathrm{new}\;}\right)$ into experience replay buffer D;12:    Sample a mini-batch of β transitions $\left(o^{m},a^{m},r^{m},o_{\mathrm{new}}^{m}\right)$ from replay. Buffer D;13:    Set $y^{m}=R^{m}+{\left.\gamma Q_{i}^{\mu^{\prime }}\left(o_{\mathrm{new}}^{m},a_{1}^{\prime },\ldots ,a_{N}^{\prime }\right)\right|}_{a_{i}^{\prime }=\mu_{i}^{\prime }\left(o_{i}\right)}$
14:    Update critic by minimizing the loss     
$L\left(\theta_{i}\right)=\frac{1}{S}\sum_{m=1}^{S}{\left(y^{m}-Q_{i}^{\mu }\left(o_{\mathrm{new}\;}^{m},a_{1},\ldots ,a_{N}\right)\right)}^{2}$
15:    Update actor using the sampled policy gradient:$\nabla_{\theta_{i}}J=\frac{1}{S}\sum_{m=1}^{S}{\left.\nabla_{\theta_{i}}\mu_{\theta_{i}}\left(o_{i}^{m}\right)\nabla_{a_{i}}Q_{i}^{\mu }\left(o^{m},a_{1},a_{2},\ldots ,a_{N}\right)\right|}_{a_{i}=\mu_{\theta_{i}}\left(o_{i}^{m}\right)}$16:    Updating actor networks             Updating critic networks             Update target networks with updating rate τ:            $\theta_{i}^{\mu^{\prime }}\leftarrow \theta_{i}^{\mu }+(1-\tau )\theta_{i}^{\mu^{\prime }}$            $\theta_{i}^{Q^{\prime }}\leftarrow \theta_{i}^{Q}+(1-\tau )\theta_{i}^{Q^{\prime }}$17:  **end for**18: **end for**

Now we resort to the MADDPG algorithm that extends DDPG to the multi-agent scenario. Specifically, a multi-agent game with L agents, $\mu_{\theta }=\left\{\mu_{\theta_{1}},\ldots ,\mu_{\theta_{L}}\right\}$, which is parameterized by $\theta =\{\theta_{1},\ldots ,\theta_{L}\}$, can be regarded as the set of all deterministic policies for all agents, the gradient of the expected return for each agent is expressed as

(15) $$\nabla_{\theta_{i}}J\left(\mu_{\theta_{i}}\right)=E_{G,a\sim D}\left[\nabla_{\theta_{i}}\mu_{\theta_{i}}\left(a_{i}|s_{i}\right)\times {\left.\nabla_{a_{i}}Q_{i}^{\mu }\left(o,a_{1},a_{2},\ldots ,a_{L}\right)\right|}_{a_{i}=\mu_{\theta_{i}}\left(s_{i}\right)}\right]$$

where $Q_{i}^{\mu }\left(o,a_{1},a_{2},\ldots ,a_{L}\right)$ is a centralized function with the inputs of all the agents’ action $a=\left\{a_{1},\ldots ,a_{L}\right\}$, as well as the observation information o. $G=\left\{o_{1},o_{2},\ldots ,o_{L}\right\}$ represents the information that the environment feeds back to the agent, including the distance between agents, the distance between agent i with surrounding obstacles, and the distance from agent i to each targets, all of which constitute the observation of all agents that are denoted by $\left[x,y,d_{i}^{U},d_{i}^{O},d_{i}^{T}\right]$. In our networks, the inputs of the critic network are the sates of all the USVs, the distance between USVs, as well as distance from the USV to the target area. The output of the critic network, i.e., $Q_{i}^{\mu }(o,a)$ is returned to the actor network for evaluating the action, which can be seen in Figure 2. The actor i executes the action based on its own policy $\mu_{\theta_{i}}$, and the critic uses the $Q_{i}^{\mu }$ function to evaluate the action of the actor. In order to remove the correlation of the samples generating from the environment, the framework adopts the experience replay mechanism to store the experience of each agent, which is a finite buffer with capacity M expressed as the tuple $\left(o,o^{\prime },a_{1},\ldots ,a_{L},r_{1},\ldots ,r_{L}\right)$. When the replay buffer is full, the oldest samples will be discarded. In order to better adapt to the environment and improve the stability of exploration, we updated the target network in a “soft update” way, so that it can slowly track the learning strategy, which can be expressed as $\theta^{\prime }\leftarrow \tau \theta +(1-\tau )\theta^{\prime }$. In each time step, a batch size of tuples was selected to train the network, and the critic-network was updated by minimizing the loss function as

(16) $$L\left(\theta_{i}\right)=E_{o,o^{\prime },a,r}\left[{\left(Q_{i}^{\mu }\left(o,a_{1},\ldots ,a_{L}\right)-y\right)}^{2}\right]$$

where

(17) $$y=r_{i}+{\left.\gamma Q_{i}^{\mu^{\prime }}\left(o^{\prime },a_{1}^{\prime },\ldots ,a_{L}^{\prime }\right)\right|}_{a_{i}^{\prime }=\mu^{\prime }\left(o_{i}^{\prime }\right)}$$

The update policy of the actor-network is expressed as

(18) $${\left.\nabla_{\theta_{i}}J\approx \frac{1}{m}\sum_{k}\nabla_{\theta_{i}}\mu_{i}\left(o_{i}^{k}\right)\nabla_{a_{i}}Q_{i}^{\mu }\left(o^{k},a_{1}^{k},\ldots ,a_{L}^{k}\right)\right|}_{a_{i}=\mu_{i}\left(o_{i}^{k}\right)}$$

where m and k represent the minibatch size and the experience index, respectively.

## 4. Simulation Results

In this section, we present the simulation results as well as the performance analysis of the proposed algorithm. In Section 4.1, the settings for the simulation are developed. Section 4.2 presents the algorithm training configurations. In Section 4.3, we present the detailed figures for the reward, collision rate, and trajectories.

### 4.1. Environment Settings

We consider a 400 * 400 square training environment based on the open AI platform, which consists of USVs, targets, and threat areas. The geometric coordinate system is established with the environmental center as the origin of the coordinates. As the target area and static obstacle are regarded as parts of the environment, their settings would not be changed during the training process. In contrast, the dynamic obstacle changes position randomly after each training episode. In our settings, the maximum communication distance $\rho_{c}$ is set to 0.9 and the width of critical area σ is set to 0.1. In this coordinate system, the size of the threat area is set to 0.25, while the size of the agent and target area are set to 0.04 and 0.20, respectively. We present two indicators to measure the effectiveness of training: the collision rate of USVs’ collisions during the current episode and the collision rate between the agent and the critical area during the total episode.

### 4.2. Training Configuration

In the given scenario, we set 60 episodes with 1000 steps each during the training process. As the mission is continuous, we manually aborted the training process in each training, and the environment was reset automatically after each episode. Since the capacity of the memory buffer we set was 3000, the training process did not start until the buffer was full, thus each USV adopted a random policy in the first 3000 steps, and then the whole network started to be trained in each step afterward.

The whole training networks of the mission planning for the AUV are constructed based on the PyTorch framework, where the training process is implemented on the NVIDIA GeForce RTX 3060 GPU. The critic network consists of five fully connected layers, including three hidden layers, of which the hidden units are set as 64, 52, and 30, respectively. The ReLU function is adopted as the non-linearity function in the networks. Specifically, the first hidden layer with 64 neurons obtains the concatenated vector of observations o and actions a, and the data then proceed through the following second and third hidden layers, with 52 and 30 neurons, respectively. The whole process is offline and can be divided into two parts: off-line centralized training and on-line decentralized execution. The advantage of using this method is that the system will have competitive execution speeds compared to the traditional methods during the testing stage, and the calculation could be gathered in the training stage.

### 4.3. Simulation Figures

**Figure 3:**
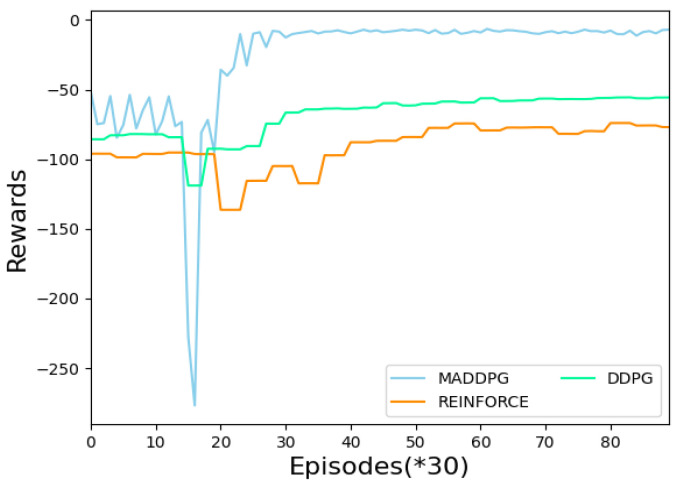
Rewards versus episodes under different RL algorithms.

Figure 3 shows the episode rewards obtained by USVs under different algorithms. The RL algorithm based on the policy gradient is used as the benchmark comparison algorithm. The experimental setting is for two USVs to perform navigation and assignment tasks with two target areas under the condition of no obstacles. It can be seen that the MADDPG has a significant advantage over the DDPG and REINFORCE algorithms, as it can get a higher reward, which proves the effectiveness of the proposed algorithm in solving multiple USVs’ navigation and area allocation tasks.

**Figure 4:**
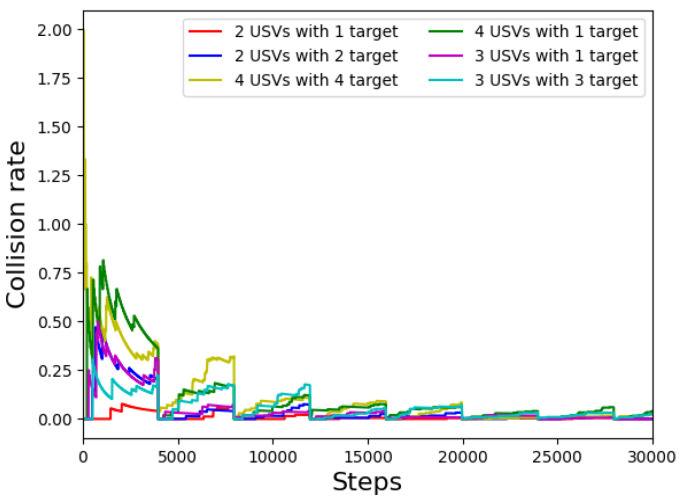
Collision rate per episode for the different numbers of USVs.

**Figure 5:**
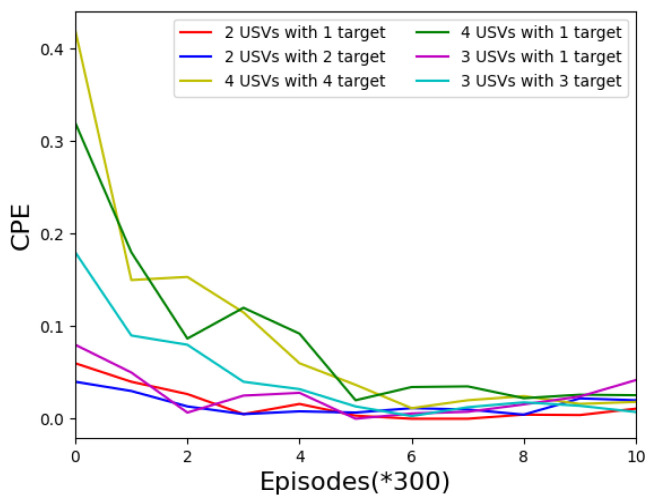
Collision rate in total episodes for the different numbers of USVs under the MADDPG algorithm.

Figure 4 shows the collision rate of USVs in different task environments. We observe the experimental effects when the number of USVs is 4, 3, and 2, and the number of target areas is 1, 2, 3, and 4, respectively. It can be seen from the figure that with the increase in training times, the collision rate of USVs in each turn will gradually decrease and tend to converge. At the same time, the total number of collisions will gradually decrease and stabilize, as can be seen in Figure 5.

**Figure 6:**
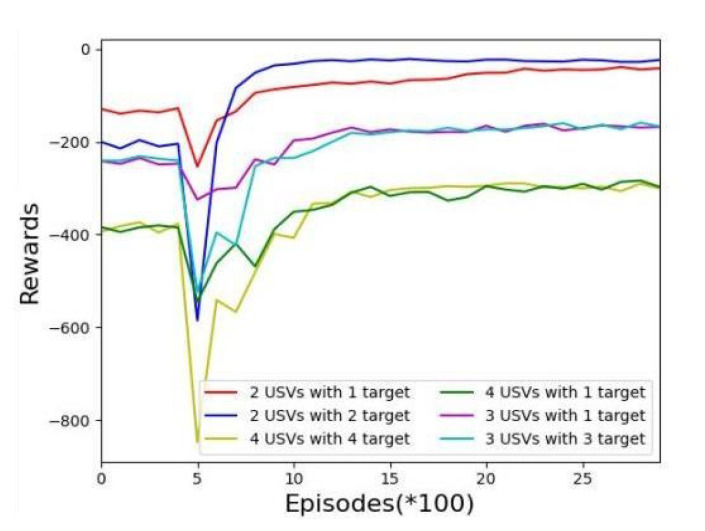
Rewards versus episodes for the different numbers of USVs under the MADDPG algorithm.

Figure 6 shows the experimental conditions of the USV reward. It can be seen that in the early stage of the training, because of the blindness of the exploration stage, the reward for each USV will undergo a dramatic decline in the lowest (about 500 rounds), but with ongoing exploration, the neural network with gradually learn the best strategy.

**Figure 7:**
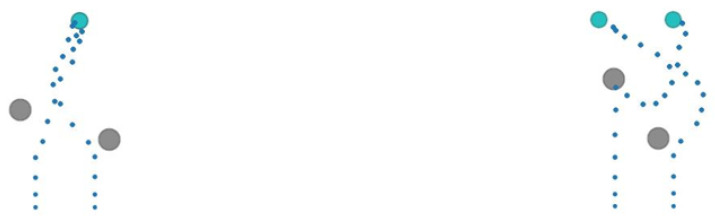

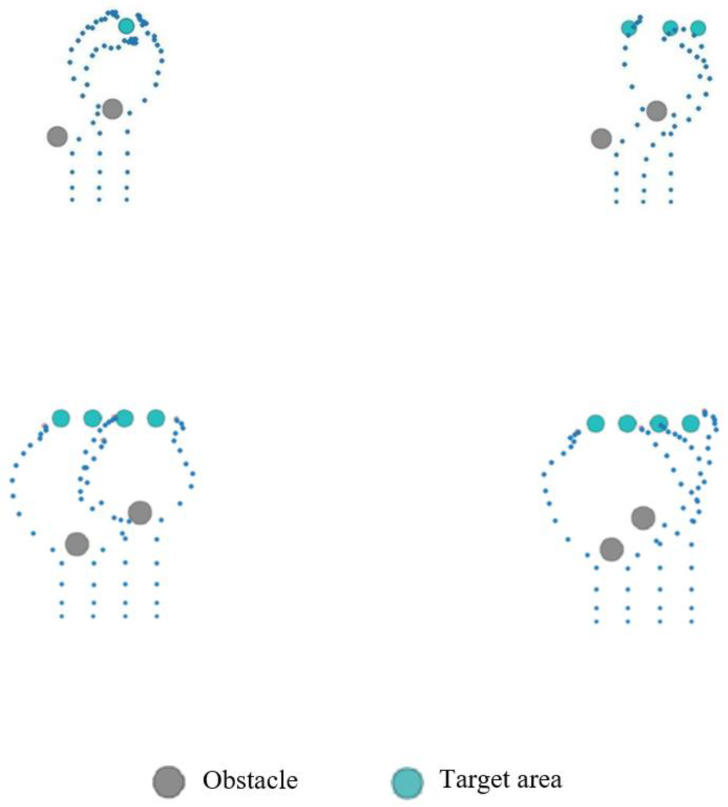
Dynamic trajectories of multi-USV.

Figure 7 shows the dynamic trajectory of USVs in different test environments. Obstacles will move randomly after each training round, while the position of the target area remains unchanged. The number of USVs and target areas can be set manually. When encountering obstacles, it will adjust the direction according to the security of the surrounding environment and the position of the target area. When approaching the target area, it will automatically coordinate the area to be reached according to the position of each target area to complete the task assigned by the area.

## 5. Conclusions

In this paper, a multi-agent reinforcement learning framework is proposed to solve the trajectory design and area assignment for the multi-USV cooperation scenario. In this framework, each USV needs to select the closest target area so as to obtain the shortest route, during which each USV should keep a certain distance from others to keep within the communication distance and avoid collisions. All UAVs use the MADDPG to try to find the optimal policy to obtain a better reward, while traditional optimization methods can hardly handle the scenario with a large number of UAVs. Thus, the proposed method can be applied to the trajectory design and area assignment for multi-UAV scenarios. The simulation results show the effectiveness of the proposed framework.

## Data Availability

Not applicable.
